# Dietary Medium Chain Fatty Acid Supplementation Leads to Reduced VLDL Lipolysis and Uptake Rates in Comparison to Linoleic Acid Supplementation

**DOI:** 10.1371/journal.pone.0100376

**Published:** 2014-07-21

**Authors:** Daniël B. van Schalkwijk, Wilrike J. Pasman, Henk F. J. Hendriks, Elwin R. Verheij, Carina M. Rubingh, Kees van Bochove, Wouter H. J. Vaes, Martin Adiels, Andreas P. Freidig, Albert A. de Graaf

**Affiliations:** 1 TNO, Zeist, the Netherlands; 2 Analytical Sciences division, The Leiden Amsterdam Centre for Drug Research, Leiden, the Netherlands; 3 The Netherlands Bioinformatics Centre (NBIC), Nijmegen, The Netherlands; 4 Department of Mathematical Sciences, University of Gothenburg, Gothenburg, Sweden; University of Catania, Italy

## Abstract

Dietary medium chain fatty acids (MCFA) and linoleic acid follow different metabolic routes, and linoleic acid activates PPAR receptors. Both these mechanisms may modify lipoprotein and fatty acid metabolism after dietary intervention. Our objective was to investigate how dietary MCFA and linoleic acid supplementation and body fat distribution affect the fasting lipoprotein subclass profile, lipoprotein kinetics, and postprandial fatty acid kinetics. In a randomized double blind cross-over trial, 12 male subjects (age 51±7 years; BMI 28.5±0.8 kg/m^2^), were divided into 2 groups according to waist-hip ratio. They were supplemented with 60 grams/day MCFA (mainly C8:0, C10:0) or linoleic acid for three weeks, with a wash-out period of six weeks in between. Lipoprotein subclasses were measured using HPLC. Lipoprotein and fatty acid metabolism were studied using a combination of several stable isotope tracers. Lipoprotein and tracer data were analyzed using computational modeling. Lipoprotein subclass concentrations in the VLDL and LDL range were significantly higher after MCFA than after linoleic acid intervention. In addition, LDL subclass concentrations were higher in lower body obese individuals. Differences in VLDL metabolism were found to occur in lipoprotein lipolysis and uptake, not production; MCFAs were elongated intensively, in contrast to linoleic acid. Dietary MCFA supplementation led to a less favorable lipoprotein profile than linoleic acid supplementation. These differences were not due to elevated VLDL production, but rather to lower lipolysis and uptake rates.

## Introduction

The type of fatty acids consumed as dietary fats is known to influence risk factors for cardiovascular disease [1]. Medium-chain fatty acids (MCFAs), being eight to ten carbon atoms long, are used as dietary supplements in weight-loss programs, since they were frequently found to lead to greater weight loss than dietary long-chain fatty acids [2]. However, MCFAs were frequently found to increase fasting plasma cholesterol and triglycerides in comparison with long-chain triglycerides [3]. Linoleic acid (C18:2n-6), which is found in several vegetable oils, can be used for cholesterol lowering when used in considerable quantities [4].

Dietary MCFA and linoleic acid undergo processing via distinct metabolic routes. MCFA, after being absorbed by the intestine, is mostly transported through the portal vein to the liver as free fatty acid. In the liver it is packaged in VLDL lipoproteins and distributed further to other target organs [3]. On the other hand, linoleic acid is generally packaged in large chylomicron particles in the intestine; from there it proceeds directly through the blood to any target organ [5]. Therefore both the role of the liver in the metabolic route and the type of particle used for transport are different for the two types of fatty acid.

Next to chylomicrons and VLDL, lipoprotein classes include the successively smaller and denser IDL, LDL and HDL particles. The VLDL particles that the liver produces are delipidated by extrahepatic tissues in a process called lipolysis. This process progressively diminishes the particle's size, which first become smaller VLDL, then IDL and finally LDL particles [6]. The LDL particles have little triglyceride left, they mainly contain cholesterol and also HDL particles' core mainly consist of cholesterol [7]. So although the role of LDL and HDL in fatty acid metabolism is limited, LDL may be a rest product of an upregulated VLDL production. Because MCFA are transported from the intestine to the liver directly and are there packaged into VLDL, it is intuitive to expect that VLDL production is upregulated when MCFA is supplemented in the diet. Since linoleic acid does not necessarily pass the liver before being transported to other tissues, supplementing the diet with this fatty acid is not expected to upregulate VLDL production in the liver much. According to this mechanism, MCFA supplementation is therefore hypothesized to result in higher rates of VLDL production than linoleic acid supplementation.

The second mechanism that is able to affect lipoproteins is PPAR-activation by linoleic acid [8]–[10]. Fibrates, also PPAR activators, are known to increase LPL lipolysis and increase liver uptake of LDL particles [11], [12]. The response to fibrates is heterogeneous and depends on the dyslipidemic state of the subject [13], [14]. It is therefore interesting to see which of the two mechanisms, upregulated VLDL production after MCFA supplementation or upregulated VLDL lipolysis and uptake after linoleic acid supplementation, are the strongest determinant for the difference in blood lipids after these two interventions.

Dietary fatty acids, transported by lipoproteins, reach various types of tissue. The tissue that eventually stores the fat is relevant, since subcutaneous and visceral adipose tissue have different metabolic activities and have different implications for cardiovascular disease risk; visceral adipose tissue is thought to confer most risk (see e.g. [15]). As a crude measure of this risk, either the waist-hip ratio (WHR) or the waist circumference are often used [16].

In this study we investigated how dietary fatty acids and body fat distribution affect lipoprotein metabolism. More specifically, we studied how dietary MCFA and linoleic acid supplementation and body fat distribution affect the fasting lipoprotein subclass profile, lipoprotein kinetics, and postprandial fatty acid kinetics. For this purpose we conducted a randomized double blind cross-over trial. We were especially interested to see which of the metabolic processes affecting VLDL was important for the expected difference in VLDL concentration between MCFA and linoleic acid supplementation.

## Materials and Methods

### Ethics Statement

This study was approved by the Medical Ethics Committee of Tilburg (METOPP) (April 18, 2007) and conducted according to the current assembly (52nd) of the Declaration of Helsinki (Edinburgh, Scotland, October 2000) and the ICH Guidelines for Good Clinical Practice (ICH Topic E6, adopted 01-05-1996 and implemented 17-01-1997).

### Study design and subjects

The study was conducted at TNO, where subjects were recruited from a pool of volunteers. After being informed about the study, both verbally and in writing, each subject signed the informed consent form. Twelve healthy male subjects aged between 30 and 60 years and with BMI between 27 and 35 kg/m^2^ were included in the study. Subjects were selected so that range of waist-hip ratio (WHR) was as high as possible, in the selected group WHR ranged from 0.93 to 1.12. Subjects with WHR<1 (n = 7) were considered lower body obese (LBO) and subjects with WHR>1 (n = 5) upper body obese (UBO) [17]. The baseline characteristics of these subject groups can be found in **Table 1**. Habitual spread intake was asked prior to the study. Subjects were used to consuming margarine and were non-restrained eaters, scoring lower than <3.25 on the Dutch Restrained Eating Behavior Questionnaire [18].

**Table 1:** Baseline characteristics of the 12 male subjects (mean ± sd).

Parameter	*UBO (n = 5)*	LBO (n = 7)
Age (yrs)	48±8	52±6
BMI* (kg.m^−2^)	28.7±0.8	28.4±0.9
Waist (cm)	105±3	99±3
WHR	1.04±0.05	0.97±0.03
DEBQ**	2.4±0.7	2.4±0.5
Body fat (MRI)	37.4±7.6	34.4±6.0
Glucose (mmol/L)	6.0±0.1	5.7±0.5
Insulin (mU/L)	8.2±1.9	9.6±5.2
Total Cholesterol (mmol/L)	5.3±0.6	5.7±1.0
LDL-cholesterol (mmol/L)	3.2±0.4	3.8±0.9
HDL-cholesterol (mmol/L)	1.5±0.3	1.2±0.2
Triglycerides (mmol/L)	1.2±0.5	1.5±0.5

^*^BMI  =  Body Mass Index: is the ratio between the body weight (kg) and the square of the height in meters of a person and is a (healthy) weight index.

^**^Dutch Eating Behavior Questionnaire for assessment of restrained, emotional, and external eating behavior.

The study was designed as a randomized, double-blind, cross-over trial and two treatments were supplied for 3 weeks with a wash-out period of 6 weeks in-between. The two dietary interventions consisted of consumption of a spread containing predominantly long chain fatty acids (71% long-chain fatty acids (mainly C18:2), 28% C16:0 and 1% fatty acids shorter than C16) and consumption of a spread containing predominantly medium chain fatty acids (65% C8:0 and C10:0, 29% C16:0 and 6% fatty acids longer than C18). In both spreads a similar percentage palmitic acid (C16:0: 28–29%) was present, necessary to make a margarine spreadable.

The treatments were given in random order. Randomization was restricted for age, BMI and WHR, resulting in a homogeneous distribution of these parameters among treatments. During the start of the intervention period subjects consumed 30 grams of test spread daily to become habituated to the fatty acids; during the rest of the study subjects consumed 60 grams of the spread daily for 2.5 weeks. The test spread replaced their normal spread and oil use. Two portions of 30 grams of spread were consumed, one with each main meal. With the test spread provided, about 50% of the daily fat intake was of experimental origin. Compliance with the spread consumption prescription was closely monitored throughout the study. Of the 960 spread consumption moments in the study only 12 consumptions were forgotten, therefore compliance was very good (98.8%).

On average [19] the male population in the studied age group consumes in total about 105 grams of fat daily, distributed as follows: about 11 grams of fat is consumed with breakfast, 26 grams with lunch, 42 grams with dinner and 26 grams with snacks. Subjects used the provided spreads with breakfast and lunch and were allowed to use some with their dinner. Assuming that the average amounts of fat consumed with dinner and snacks did not change, we may expect that on an average, fat intake may have been increased with about 20 grams daily.During the complete study period (84 days) no changes were seen in body weight or waist and hip circumferences. Also, the six weeks wash-out in-between both test periods did not affect these main characteristics of the study population.

### Standardized diet and meals

**Figure 1:**
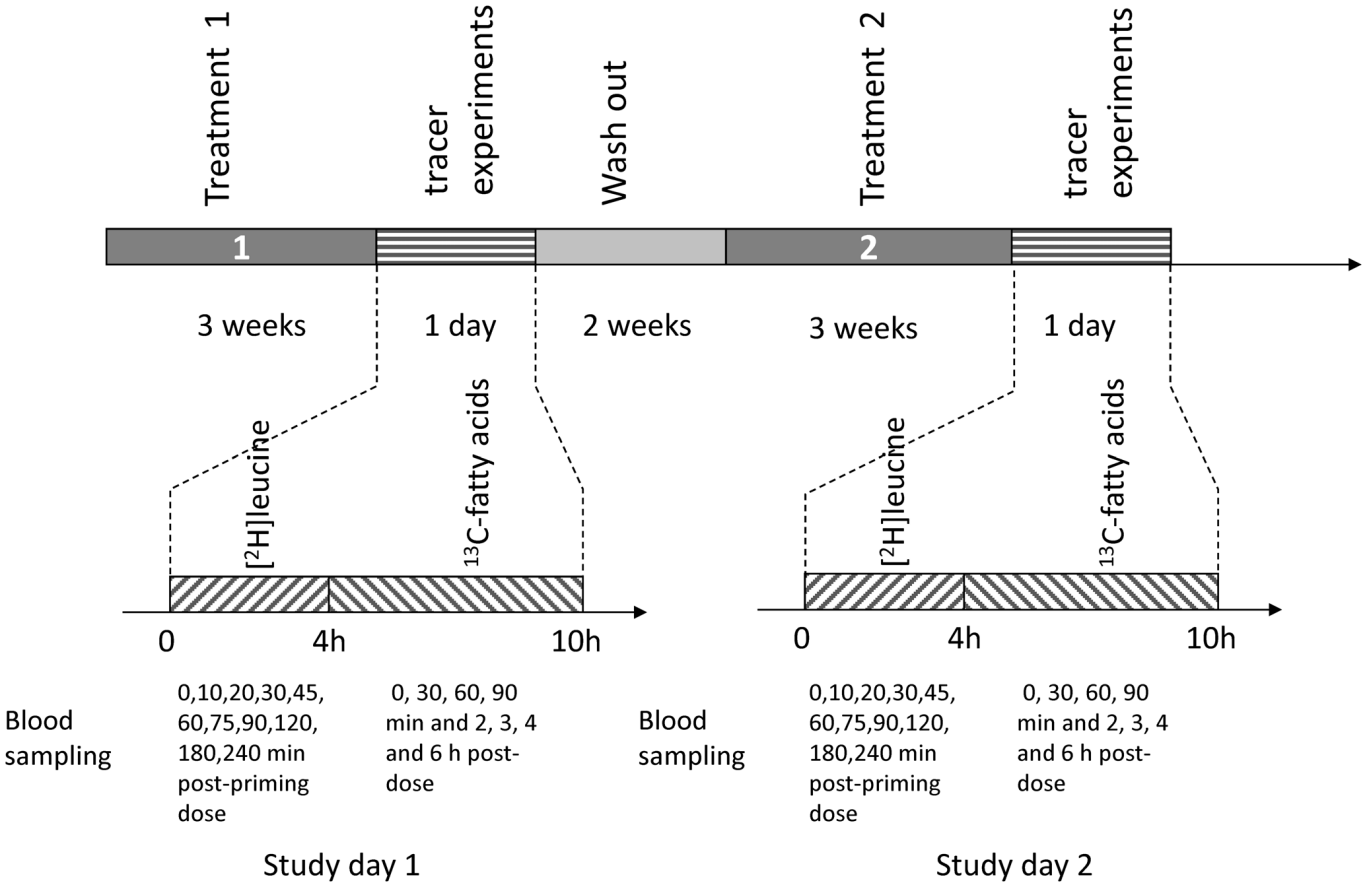
Schematic overview of the test day.

On day 20 and day 62 (day before test day) subjects ate a standardized evening dinner at home. The dinner consisted of a microwave lasagna meal and a yogurt dessert. The lasagna meal of 400 grams contained 548 kCal, 50 grams of carbohydrates, 30 grams of fat, 20 grams of protein and 5 grams dietary fiber. On day 21 (and day 63), the test days (see Figure 1 for an overview), subjects came to TNO after an overnight fast around 08:00 h. On these days subjects were not provided with a breakfast and kept fasted for the first four hours during the tracer experiment. At lunchtime subjects consumed one of the study products (spreads) with fatty acid tracers added on soft rolls. Lunch was provided in between the two tracer experiments, between 12:00 and 13:00 hours, and a snack was provided four hours after lunch.

In between breakfast and lunch subjects were allowed to drink water. Coffee or tea could be chosen on the first test day in the afternoon (with a maximum of 2 cups), but had to be the same on the other day. Compliance to consumption of the drink(s) was checked and registered.

#### Lunch

Due to the fact that subjects arrived fasted and remained fasted till 12:00–13:00, a lunch containing 40% of the total energy intake was provided. No particular choice of macronutrient composition was made. The lunch contained three brown soft rolls; 30 grams of test margarine; one slice of cheese (20 grams); three portions of marmelade (45 grams); 250 mL milk (semi-skimmed), 200 mL of orange juice and one banana. The total lunch contained about 40% of the total energy intake of a day (1068 kcal). The macronutrient composition of the lunch was: 57 En% carbohydrates; 32 En% fat; 12 En% protein.

The fatty acids stable isotopes were added to the lunch supplied.

#### Snack

The snack, four hours after lunch, consisted of a soft drink (“Fanta”) with a treacle waffle. With this snack 224 kcal was ingested. No particular choice of macronutrient composition was made. The total snack contained about 10% of the total energy intake of a day. The macronutrient composition of the snack was: 79 En% carbohydrates; 19 En% fat; 2 En% protein. Subjects were provided with (soft) rolls at the end of the day. Water was available throughout the day.

### Lipoprotein profiles

After three weeks of spread intervention, a blood sample was obtained from the subjects after an overnight fast. Lipoprotein profiles were measured by LipoSearch, which quantifies cholesterol and triglycerides levels in each of the major lipoprotein classes (chylomicrons, VLDL, LDL and HDL) as well as in 20 well defined sub-classes. Plasma samples were shipped frozen and analysed by Skylight Biotech Inc. according to Okazaki et al [20].

### Fatty acid turnover

During the experiment, the test spreads provided approximately 50% of the daily fat intake. Due to the particular fatty acids provided it was assumed that changes in physiology and fat metabolism would take place. The metabolic fluxes of fats and lipoproteins were measured using stable isotopes.

Selection of the tracers and doses of the tracers was based on prior studies investigating protein (turnover) and fat metabolism. In the present study stable isotopes of fatty acids and leucine were supplied to the subjects, to monitor the kinetics of fat metabolism and lipoprotein synthesis. The protocols used were based on publications of McCloy et al [21] for fatty acid tracing and Bilz et al.[12] as well as Van Eijk and Deutz [22] for apolipoprotein synthesis.

#### Tracers for lipoprotein metabolism

The stable isotopes were supplied during the test days. L-leucine was provided to the subjects in the morning when subjects were still in a fasted condition, intravenously. At start a prime was given, followed by a continuous infusion. At t = 0 on the study day, 0.60 mg/kg body weight [5,5,5-D3, 98%]L-leucine (Buchem BV, Apeldoorn, The Netherlands) prime was given as a bolus. Following the bolus a continuous infusion at a rate of 0.68 mg/kg body weight per hour was administered for 4 hours.

#### Tracers for fatty acid metabolism

The fatty acid tracers were given when the L-leucine protocol was finished, after four hours on the study day. In the afternoon the fatty acid tracers were supplied orally, by adding the tracers directly onto the lunch. The tracers consisted of 600 mg [U-^13^C_18_ (98%)]linoleic acid (C18:2) (Spectra Stable Isotope group, Columbia, MD, USA) and 600 mg [1,2,3,4-^13^C_4_, 99%]octanoic acid (C8:0) (Larodan Fine Chemicals AB, Malmö, Sweden). The tracer was followed for four hours.

### Fatty acid analytics

#### Chemicals

The chemicals used for fatty acid analytics were obtained from Biosolve and from Merck. From Biosolve Cyclohexane, AR; heptane, AR; heptadecanoic acid, A.R.; and absolute methanol were obtained. From Merck Borontrifluoride (BF3) in methanol, 20% (m/V), P.S.; sodium chloride, p.a.; sodium hydroxide, p.a.; and sodium sulfate, waterfree were obtained.

#### Fatty acid concentrations

The fatty acid profile in plasma samples was determined in accordance with ISO methods [23], [24]. In short, internal standard (heptadecanoic acid) was added to freeze-dried sample (1 mL). Fatty acids, (tri)glycerides, phospholipids and cholesterol esters were saponified with 2N sodium hydroxide in methanol (1 mL) in a boiling waterbath, to obtain free fatty acids. Fatty acid methyl esters (FAMEs) were obtained by derivatization with 20% boron trifluoride in methanol in a boiling waterbath. A saturated solution of sodium chloride in water (4 mL) was added and FAMEs were extracted with hexane (1 mL). Hexane was dried over sodium sulphate and analyzed on a Thermo Trace GC Ultra gas chromatograph (Thermo Finnigan, Breda, The Netherlands) with cold on-column injection (1 µl) on a Chrom-Pack SIL-88 column (0.2 µm, 0.25 mm, 50 m) (Chrompack, Middelburg, The Netherlands) and FID detection.

#### Fatty acid isotopic enrichments

For GC-C-IRMS determination of 13C isotopic enrichments, extracted FAMEs from plasma samples were separated using a gas chromatograph (model 6890; Hewlett Packard, Palo Alto, CA; inlet temperature 280°C), run at constant flow (1.2 ml/min) with splitless injection equipped with a Chrom-Pack SIL-88 column (0.2 µm, 0.25 mm, 50 m). The GC was connected to a ThermoFinnigan Delta-plus IRMS mass spectrometer (Thermo Finnigan, Breda, Netherlands) operated with the oxidation and reduction temperatures set at 960°C and 600°C respectively, with 100% CO2 as the reference gas. The isotope of the reference gas was calibrated using certified carbohydrate standards (C15H32, C20H42 en C25H52) from Chiron AS, Trondheim, Norway. The starting temperature was 50°C for 4 minutes, then the system was ramped at 10°C per minute to 160°C, thereafter ramped at 1°C per minute to 195°C and followed by a 5°C per minute increase to the final temperature 225°C, where the system was kept for 10 min.

Carbon-13 enrichment values were computed by the IRMS instrument software in units of atom percent (AP): 
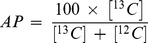



The percentage of ^13^C enrichment above baseline, expressed as atom percent excess (APE), was calculated by subtracting baseline AP from measured values at each time point (t) for each individual fatty acid: APE  =  AP_t_ - AP_baseline_.

The concentration of ^13^C-labeled fatty acid in the plasma lipid classes was calculated by multiplying APE by the measured total concentration for each fatty acid. The percentage of dose in plasma was calculated using the tracer concentration (*[^13^C]*, in mg/liter plasma) at each time point and the tracer dose administered (*D*, in mg ^13^C - labeled fatty acid):
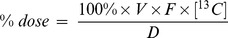



Where the factor F corrects for the incomplete ^13^C labeling of fatty acids of chain length N derived from [1,2,3,4-^13^C_4_]octanoic acid, as follows:
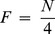
and V is the total plasma volume:
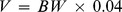
where it is assumed that the plasma volume is 40 ml per kilogram body weight. Expressing the dose in total plasma instead of per l plasma as in (McCloy et al 2004) reduces variations associated with differing plasma volumes in persons differing in body weight.

The percentage dose in plasma was calculated for each fatty acid and for each time point and was plotted against time (min). The area under the curve (AUC) until time point t = 240 min after fatty acid tracer addition was calculated to give a relative measure of the amount of label appearing over the 4 h period of the study. Since APE values had not reached a plateau at t = 240 min, the APE at t = 240 was taken as peak enrichment. The % dose values at t = 240 were used as a measure of the speed of ^13^C label incorporation.

In addition to the tracers C8:0 and C18:2n-6, isotopically enriched fatty acids C10:0, C12:0, C14:0, C16:0, C18:0 and C18:1 were found in the plasma samples. Because we reasoned that these were metabolically derived from [1,2,3,4-^13^C_4_]C8:0 by chain elongation *in vivo*, we also calculated the enrichment of the total C8:0 plus all fatty acids that underwent chain elongation, and compared that to C18:2n-6 enrichment. The additional carbon recruited by fatty acid chain elongation to arrive at chain length N was calculated and expressed in percentage dose equivalents for each time point as:
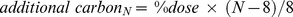



The contributions of C10:0, C12:0, C14:0, C16:0, C18:0 and C18:1 were added to arrive at the total amount of additional carbon recruited for chain elongation in units of percentage dose at each time point. From the values at each time point, the AUC until time point t = 240 of additional carbon recruitment was calculated.

### ApoB analytics

#### Chemicals

The following chemcials were used: acetonitrile (“HPLC-S”grade, from Biosolve), water (MilliQ, from Millipore), formic acid 98–100% pa (from Merck), hydrochloric acid 37% pa (from Merck), leucine >99% (from Fluka), 15N-leucine 98% atom 15N (from Isotec), 2H3/d3-leucine (methyl-d3) 99% atom 2H (from CDN Isotopes), apolipoprotein B from human plasma, ∼95% (from Sigma).

#### Plasma lipoprotein profiling by FPLC

To determine the lipid distribution over plasma lipoproteins, lipoproteins were separated using fast protein liquid chromatography (FPLC). 50 µL of plasma were injected onto a Superose 6 PC 3.2/30 column (ÄKTA System, Amersham Pharmacia Biotech, Piscataway, NJ, USA) and eluted at a constant flow rate of 50 µL/min in PBS, 1 mM EDTA, pH 7.4. Fractions of 50 µL were collected and assayed for cholesterol [25].Plasma total cholesterol was measured by an enzymatic procedure using kit no. 1489437, from Roche Diagnostics, Mannheim, Germany.

#### Hydrolysis of VLDL protein

Acid hydrolysis was applied to convert VLDL protein, i.e. ApoB, to amino acids. A volume of 90 µl of VLDL solution was mixed with 20 µl 15N-leucine solution (2.5 µg/ml in water) and 120 µl hydrochloric acid (37%) in a 1.5 ml glass vial (from Grace). Capped vials were placed in a stove at 120°C for 20 hours. After hydrolysis the liquid was evaporated (N2, 60°C). The residue was dissolved in 100 µl water and transferred to an autosampler vial insert.

#### LC-MS analysis of d3-leucine and leucine

Deuterated leucine was measured with ESI LC-MS on a high resolution LTQ-Orbitrap instrument (Thermo, San Jose, USA) and an Accela HPLC-autosampler System (Thermo).

The mobile phases A and B consisted of 0.1% formic acid in water/acetonitrile (A = 0% acetonitrile, B = 80% acetontrile v/v). A simple gradient at a flow rate of 0.4 ml/min was used: 4 min at 100% A, then linear to 100% B in 5 min, followed by 5 min equilibration at 100% A. Leucine was separated with a Synergi Hydro-RP 80A 4 µm, 75×3 mm i.d. column (Phenomenex) at slightly elevated temperature (30°C). This HPLC method gives baseline separation of isoleucine and leucine. The injection volume was 10 µl.

The LTQ-Orbitrap instrument was operated in positive mode at a mass resolution setting of 30000. The instrument was scanned from m/z 131 to 137 to measure leucine (m/z 132.1019), 15N-leucine (m/z 133.0989), and d3-leucine (m/z 135.1207). The 15N labeled leucine was used as an internal standard. At 30k resolution setting the 15N and d3 isotopes are well resolved from other natural isotopes (e.g. 13C, 17O and 18O). The natural abundance of the 15N isotope of leucine is only 0.37%, and the amount of 15N leucine was selected to have a minimal contribution of the 15N isotope from leucine from ApoB leucine.

#### Determination of ApoB amount in VLDL fractions

The VLDL hydrolysates were analyzed together with ApoB standards (0–0.5 mg/ml, also hydrolyzed with a procedure identical to the VLDL fractions). The leucine over 15N-leucine response ratio was used to calculate the amount of ApoB in the VLDL samples.

*Determination of d3-leucine enrichment (%E).* The d3-leucine enrichment was expressed as the d3-leucine and leucine peak area ratio in the VLDL samples.

### Modeling

#### Lipoprotein profiles

Lipoprotein profiles were analyzed using the previously described Particle Profiler model [6], [26]–[28]. This study constitutes the first application of Particle Profiler to HPLC lipoprotein profiles. The necessary data processing and adjustments in the data fitting process are described in File S1.

#### Apo B kinetics

The measurements of plasma leucine concentration and enrichment of free leucine in plasma and leucine in VLDL, the pool sizes of leucine (i.e., derived from apoB) in VLDL, and the known injected amounts of labeled leucine were used to determine kinetic parameters using the modeling software SAAMII (SAAM Institute, Seattle, WA).

**Figure 2:**
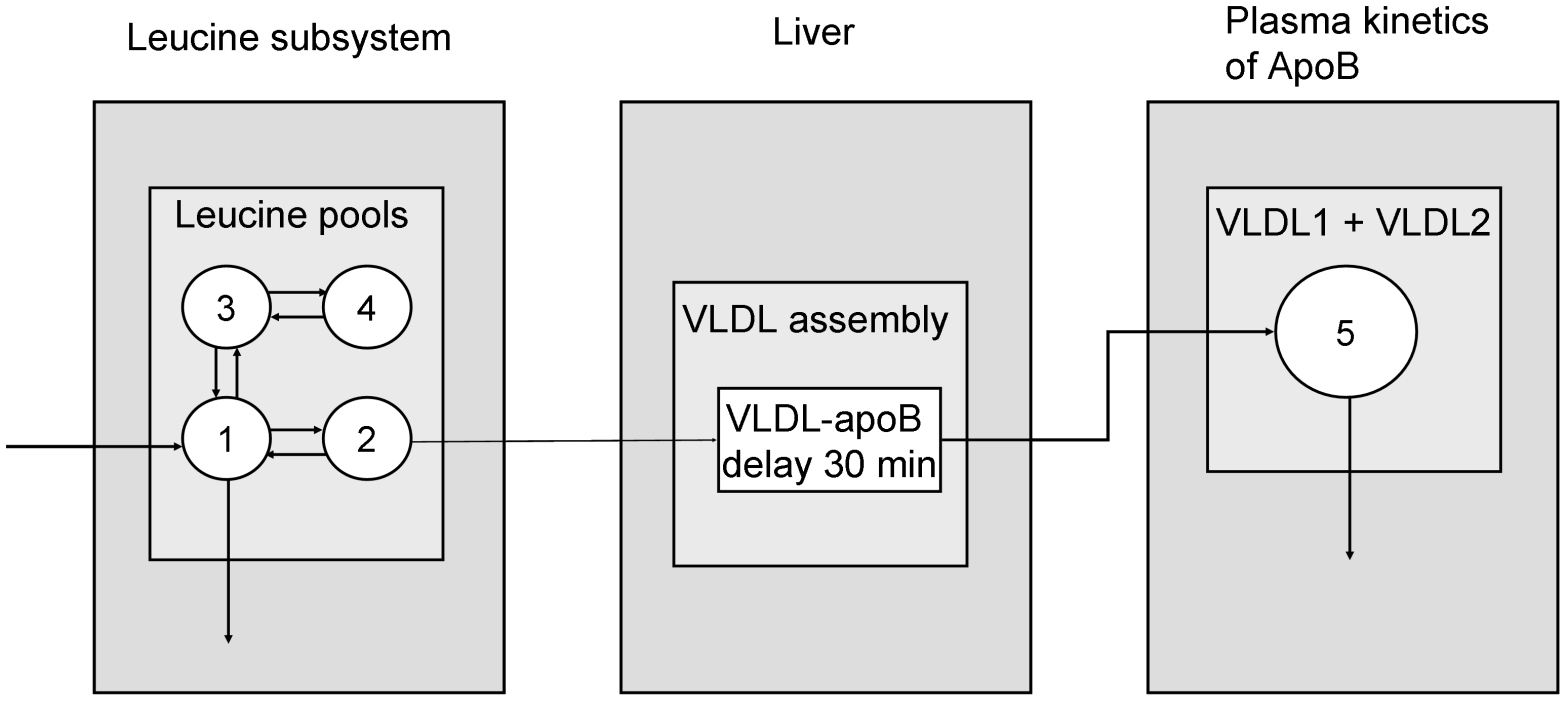
Schematic model of the compartmental model used for apolipoprotein B-100 (apoB) kinetic analysis. The assembly of lipoprotein is modeled by 9-compartment delay for apoB. The plasma apoB kinetic is modeled by a single hydrolysis step. Only a single VLDL lipoprotein fraction is considered, consisting of the VLDL1 and VLDL2 fractions in Adiels et al [29]. The free leucine plasma kinetics is modeled by two pools (3 and 4) and a plasma compartment (1), which interchange materials with an intracellular compartment (2). Compartment 2 feeds the apoB synthetic machinery.

The data were analyzed a linear compartmental model (Figure 2) that is a reduced version of the two layer apoB/TG model introduced by Adiels *et al.* [29]. Basically, the model consists of three parts: plasma leucine, the assembly of lipoprotein, and lipoprotein plasma kinetics. ApoB-leucine in a single total VLDL pool (compartment 5) was considered. The delay involved with liver VLDL assembly was fixed at 30 min. It was assumed that no dilution of hepatic L-leucine by unlabeled sources (e.g., protein degradation) took place. In other words, only the apoB synthesis from plasma leucine was considered. Free plasma leucine was modeled as a four-compartment catenary system (Figure 2). Compartment 1 is the plasma compartment, where the leucine is injected. Compartments 3 and 4 are protein pools, which give a slow release of leucine. Compartment 2 is an intracellular compartment from which the leucine is transferred into the liver's apoB synthetic machinery. The transfer coefficients between compartments 1 and 2 are equal, giving equilibrium. To further decrease the number of unknowns, k_3,4_ was set at 0.1× k_4,3_ (i.e., the transfer from compartment 4 to 3 is one-tenth of the transfer from compartment 3 to 4).

From the calculated solution to the model, the production of apoB was calculated as mg/day/kg body weight.

### Statistics

All data are expressed as mean and standard deviation. Treatment and body fat distribution effects were evaluated with two-way ANOVA. In all statistical tests performed, the null hypotheses (no effect) were rejected at the 5% level of probability (α = 0.05). Statistical analysis of the data was carried out based using the SAS statistical software package (SAS/STAT Version 8.2, SAS Institute, Cary, NC).

## Results

### Lipoprotein profiles

**Figure 3:**
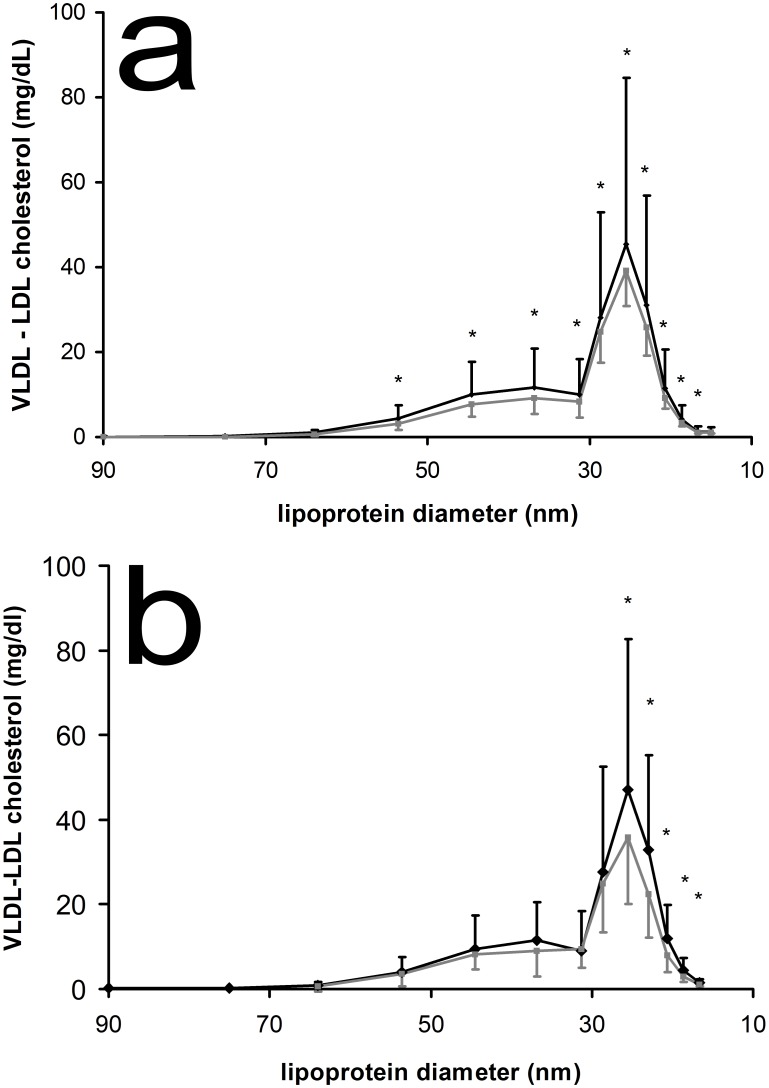
Average lipoprotein cholesterol profiles, including chylomicrons through LDL, a: after dietary MCFA (black line) and linoleic acid (grey line) supplementation and b: for lower body obese (black line) and upper body obese (grey line) supplementation. * Significant difference in two-way ANOVA between dietary supplementations with p<0.05. P-value of significant changes (from left to right, subscript indicates lipoprotein diameter) in panel a: p_64_ = 0.0804; p_54_ = 0.0072; p_45_<0.0001; p_37_<0.0007; p_31_ = 0.0319; p_29_ = 0.0211; p_26_ = 0.0108; p_23_ = 0.0141; p_21_ = 0.0179; p_19_ = 0.0125; p_17_ = 0.0098. P-value of significant changes (from left to right, subscript indicates lipoprotein diameter) in panel b: p_26_ = 0.0412; p_23_ = 0.0112;p_21_ = 0.0115;p_19_ = 0.0103;p_17_ = 0.0115.

**Table 2** shows the response of the chylomicron, VLDL, LDL, and HDL fractions after dietary MCFA versus linoleic acid supplementation and the significance of the body fat distribution effect. VLDL and LDL cholesterol and triglycerides vary significantly between treatments, whereas only LDL cholesterol shows a significant body fat distribution effect. In **Figure 3a** we see how cholesterol in the VLDL through LDL fractions differed after dietary MCFA versus linoleic acid supplementation. Cholesterol concentrations were significantly higher after MCFA supplementation for all subfractions, except the two VLDL subfractions with largest particle diameter and the LDL subfraction with smallest particle diameter. In **Figure 3b** we see how cholesterol in VLDL through LDL subfractions differed in lower body obese versus upper body obese individuals. Cholesterol concentrations in five LDL subfractions, those with the smallest particle sizes, were significantly higher in lower body obese individuals. LDL cholesterol as calculated by the Friedewald formula was not significantly different between body fat distribution groups (data not shown).

**Table 2:** HPLC lipoprotein measurements after both treatments, analyzed by two-way ANOVA for treatment and waist-hip-ratio differences (mean ± sd).

	MCT		C18:2		p-value	p-value
					treatment	WHR
Total cholesterol (mg/dL)	207.75	±39.25	182.44	±30.74	0.001	0.1352
chylomicron cholesterol (mg/dL)	0.23	±0.36	0.16	±0.19	0.411	0.5150
VLDL cholesterol (mg/dL)	37.07	±9.18	28.98	±10.68	0.001	0.5114
LDL cholesterol (mg/dL)	121.37	±31.34	103.56	±21.40	0.009	0.0304
HDL cholesterol (mg/dL)	49.07	±9.23	49.75	±8.64	interaction	interaction
Total triglycerides (mg/dL)	148.31	±50.85	121.46	±40.61	0.028	0.7662
CM triglycerides (mg/dL)	1.96	±2.96	1.46	±1.92	0.495	0.5016
VLDL triglycerides (mg/dL)	109.67	±40.36	87.24	±32.07	0.024	0.9019
LDL triglycerides (mg/dL)	25.53	±7.10	22.44	±5.93	0.018	0.2376
HDL triglycerides (mg/dL)	11.16	±3.53	10.32	±3.14	0.230	0.9838

### ApoB and lipoprotein kinetics

Supporting Figure S2 in File S1 shows a typical time curves of observed d3-leucine enrichment in apoB isolated from plasma total VLDL, taken from one subject after both treatments. The relatively short time duration of the experiment of 4 h only permitted observation of the early, almost linear increase of isotope incorporation in VLDL-apoB, which does not include enough information to estimate the parameters of a complex apoB kinetics model. This is the reason why a reduced version of the apoB kinetics model was used to analyze the data. With this version of the model, the standard deviation of the fit averaged 4.3% of the fitted production value. Therefore, the single VLDL production estimate could be accurately determined.

**Figure 4:**
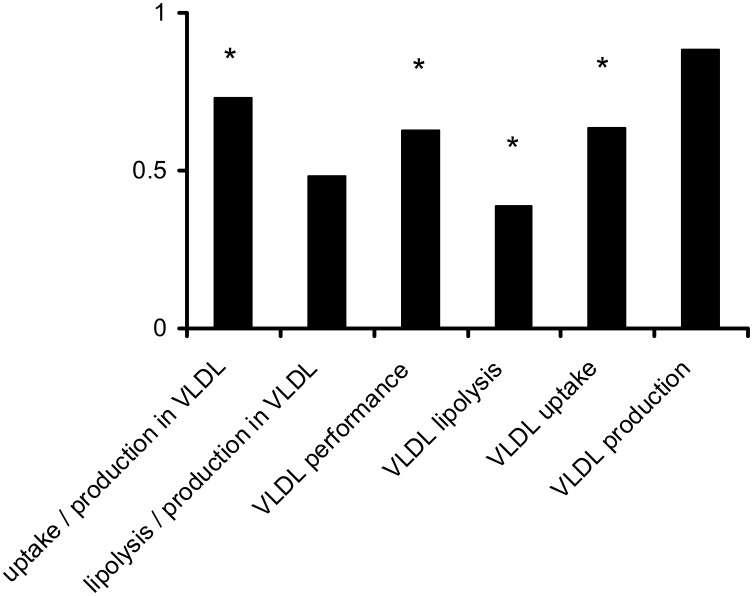
Ratios between average VLDL metabolism parameters after dietary MCFA supplementation versus linoleic acid supplementation; values < 1 indicate a higher value after linoleic acid supplementation. Ratios are shown to allow comparing differences after dietary supplementation between parameters with different dimensions. * Indicates a significant difference in two-way ANOVA between MCFA and linoleic acid supplementation, with p<0.05. The first three parameters have dimensions *volume/# particles*, the uptake and lipolysis measure have the dimension *1/time*, and the production measure has dimension # particles/(volume * time). Differences in VLDL triglyceride and cholesterol pool size can be found in table 2. The p-values of the significant measures are: uptake/production in VLDL (p = 0.030); VLDL performance (p = 0.040); VLDL lipolysis (p = 0,0213); VLDL uptake (p = 0.0135).

**Figure 4** displays the VLDL metabolism parameters derived from Particle Profiler and the compartmental modeling of VLDL production. The first three parameters shown are metabolic ratios that were calculated directly from the lipoprotein profile using Particle Profiler; they have the dimension of *volume/# particles*. VLDL performance is the average of the first two parameters. The clinical significance of these three parameters is explained by van Schalkwijk *et al.* [26]. The last VLDL production parameter was calculated directly from the apoB enrichment curves using compartmental modeling; it has dimension # particles/(volume * time).

Finally, the 4^th^ and 5^th^ parameter in the figure were obtained through multiplying the first two ratios with the VLDL production parameter; they have dimension *1/time* and can be regarded as rate constants for the average VLDL particle.

As Figure 4 shows, the uptake over production ratio in VLDL is significantly higher after linoleic acid supplementation versus MCFA supplementation. However, the lipoprotein lipase (LPL)-related lipolyis over production ratio is not significantly different between treatments. Combining the information from these ratios with the VLDL production information, we see that the difference between treatments is not due to production, but rather due to differences in LPL lipolysis and uptake; and although the VLDL LPL lipolyis over production ratio does not differ significantly between treatments, the VLDL LPL lipolysis parameter does differ significantly. Overall, this analysis indicates that through combining Particle Profiler and compartmental modeling approaches, we see that treatment differences in the VLDL lipoprotein profile are due to LPL lipolysis and uptake rather than to production. Without the combined modeling approach, this conclusion would not have been obvious.

**Figure 5:**
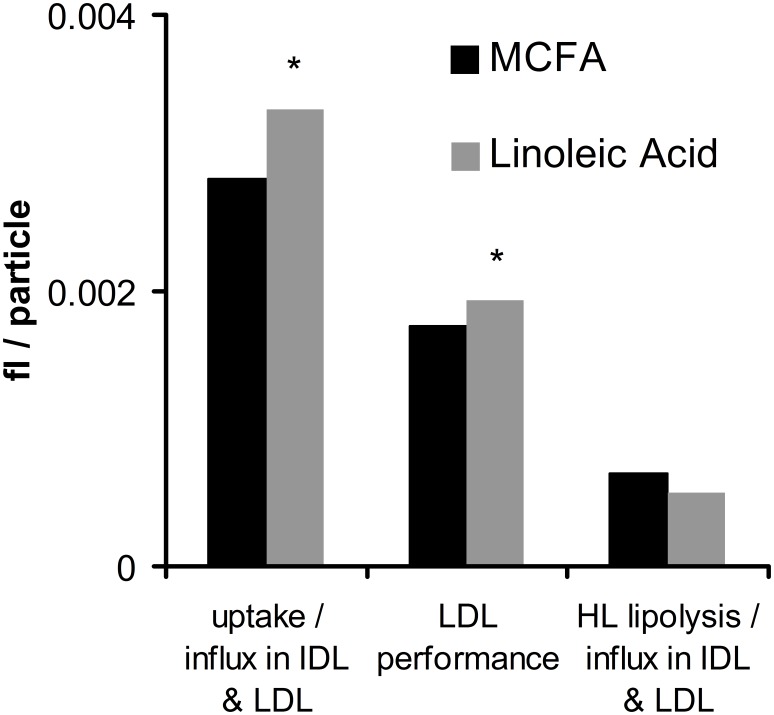
Ratios between average IDL and LDL metabolism parameters after dietary MCFA supplementation versus linoleic acid supplementation. LDL performance is the average of the two ratios shown to the sides. “fl/particle” is the unit of all three ratios. * Indicates a significant difference in two-way ANOVA between MCFA and linoleic acid supplementation, with p<0.05. p-values of the significant measures are: uptake/influx in IDL and LDL (p = 0.011); LDL performance (p = 0.010).

**Figure 6:**
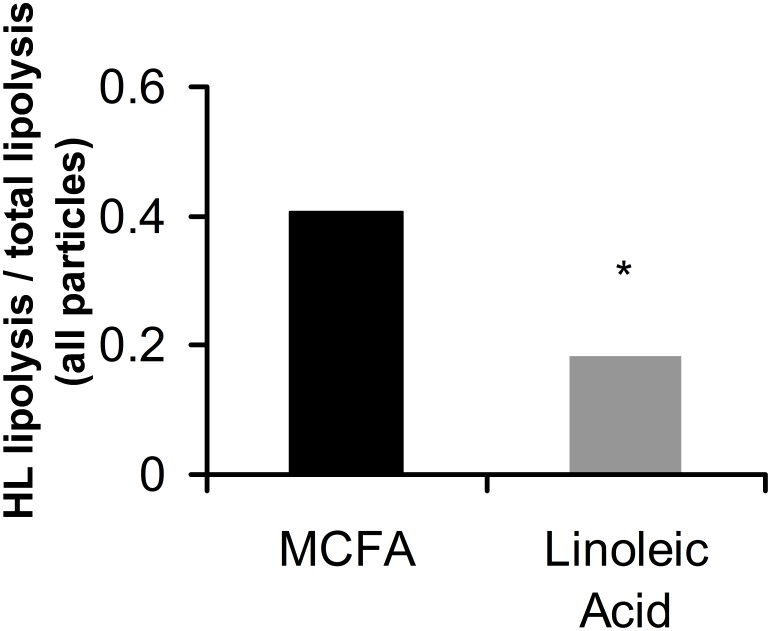
Ratios between average hepatic hipase (HL) lipolysis activity and total lipolysis activity (which also includes LPL-related lipolysis) averaged over all particles from VLDL to LDL that are included in the model, after dietary MCFA supplementation versus linoleic acid supplementation. * Indicates a significant difference in two-way ANOVA between MCFA and linoleic acid supplementation, with p<0.05 (p = 0.047).

**Figure 7:**
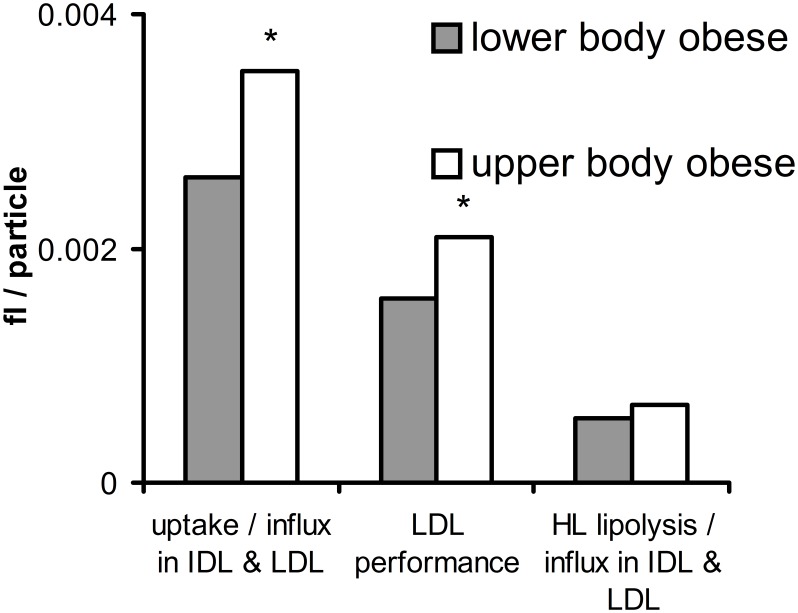
Ratios between average IDL and LDL metabolism parameters in lower body obese (LBO) versus upper body obese (UBO) subjects. LDL performance is the average of the two ratios shown to the sides. “fl/particle” is the unit of all three ratios. * Indicates a significant difference in two-way ANOVA between LBO and UBO subjects, with p<0.05. p-values of the significant measures are: uptake/influx in IDL and LDL (p = 0.012); LDL performance (p = 0.009).

Figures 5, 6 and 7 display metabolic ratios derived from Particle Profiler for the LDL to IDL region. In this region, no stable isotope data was available for quantifying absolute fluxes. The ‘influx’ referred to in several parameters is the sum of particle influx due to direct production from the liver and due to lipolysis of larger particles.

Figure 5 shows a significantly higher uptake – influx ratio in IDL and LDL after linoleic acid supplementation. The hepatic lipase (HL) lipolysis – influx ratio in IDL and LDL is not significantly different between treatments. The average of these two ratios, called ‘LDL performance’, is significantly higher after linoleic acid supplementation.

Figure 6 compares the ratios between HL-related lipolysis and the total lipolysis activity as identified by Particle Profiler; these ratios are averaged over all particles in the model. The average ratio is shown per treatment group. The figure shows that after dietary linoleic acid supplementation, the relative contribution of HL to the total lipolysis activity is lower than after MCFA supplementation. This may be due either to increased LPL activity, decreased HL activity, or a combination of these two when linoleic acid is supplemented to the diet.

Figure 7 shows a significantly higher uptake – influx ratio in IDL and LDL for upper body obese subjects. The HL lipolysis – influx ratio in IDL and LDL is not significantly different between body fat distribution categories. The average of these two ratios, called ‘LDL performance’, is significantly higher for upper body obese subjects.

### Fatty acid kinetics

Supporting Figure S3 in File S1 shows a representative example of observed time courses of ^13^C label in plasma fatty acids.

After addition of the [1,2,3,4-^13^C_4_]octanoic acid tracer, ^13^C incorporation not only in the plasma trace fatty acids (linoleic acid C18:2 and octanoic acid C8:0) but also in the saturated fatty acids C10:0, C12:0, C14:0, C16:0 and C18:0 as well as in monounsaturated C18:1 was observed. Isotopically labeled C10:0, C12:0, C14:0, C16:0 and C18:0 as well as C18:1 are all likely to sprout from the octanoic acid tracer through chain elongation. At t = 240, approximately 4% of the tracer doses of C8:0 and C18:2n-6 were present in the plasma pool. On average, 66% of the C8:0 dose appearing in plasma was present in putative elongation products of C8:0, both after MCFA and PUFA treatment. No tracer accumulation was observed in the C18:2 linoleic acid metabolic products C20:4 and C22:6.

**Figure 8:**
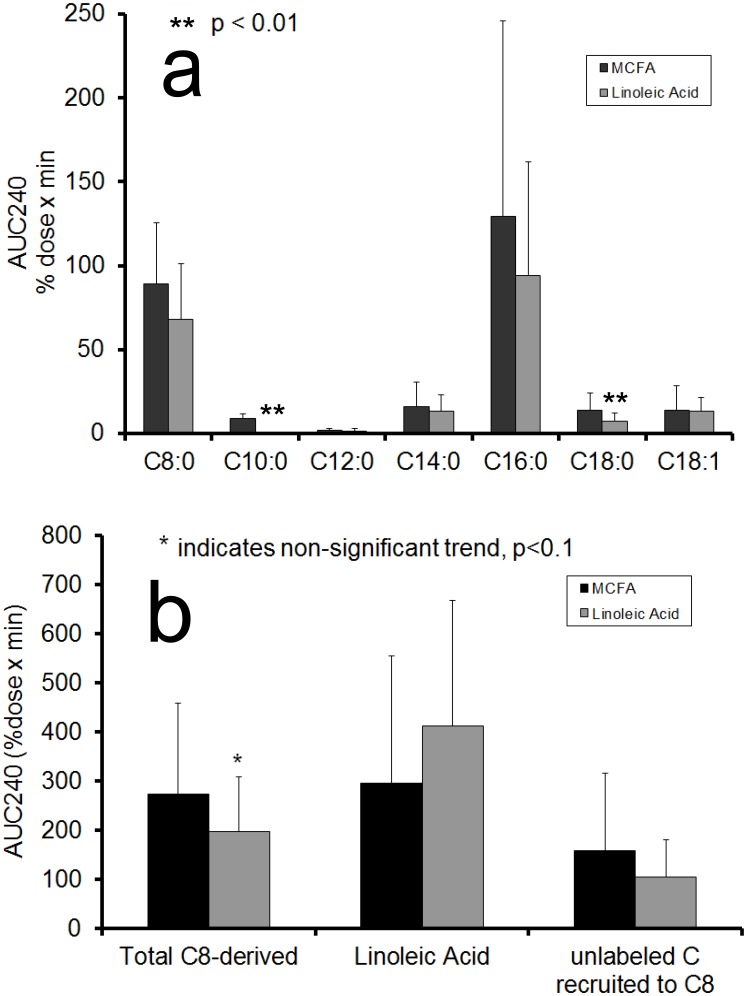
Postprandial AUC240 values (average +/− standard deviation) of % dose of the added octanoic acid and linoleic acid tracers found in plasma, compared between MCFA and Linoleic Acid dietary supplementation. Panel a: different fatty acids, p-values of the significant measures are: C10:0 (p = 0.0002); C18:0 (p = 0.0047); panel b: derived from octanoic acid, or in Linoleic Acid, or carbon recruited to elongate octanoic acid, asteriks indicates a non-significant trend.

**Figures 8a and 8b** show the results of the fatty acid kinetics analysis. Significant treatment effects were observed for C10:0, and C18:0;for the total of C8:0-derived fatty acids a non-significant trend was observed (p = 0.0812). For C8:0 a significant WHR effect was found (p = 0.0047). No WHR-treatment interactions were found. In all cases means for MCFA treatment were higher than for Linoleic Acid treatment. These results indicate a trend that a larger fraction of the MCFA dose was present in the plasma in volunteers receiving the MCFA dietary supplementation as compared to the Linoleic Acid dietary supplementation. The MCFA chain elongation recruited amounts of (unlabeled) carbon equivalent to 159+/−157%dose×min for MCT and 105+/−75%dose×min for PUFA treatment. When adding these unlabeled carbon contributions due to chain elongation to the total C8:0-dereived %dose, the result was an approximately equal amount of carbon released into the plasma for both fatty acid types.

## Discussion

### Main findings

In this study we investigated how dietary MCFA and linoleic acid supplementation and body fat distribution affect the fasting lipoprotein subclass profile, lipoprotein kinetics, and postprandial fatty acid kinetics. With respect to waist-hip-ratio differences, we found that the five smallest LDL fractions were significantly higher for lower body obese (WHR<1.0) subjects. In the lipoprotein profiles, we saw higher plasma cholesterol after MCFA supplementation than after linoleic acid supplementation, for nearly all VLDL, IDL and LDL subfractions. These differences in lipoprotein profile were found to be due to a greater VLDL lipolysis and uptake rate after linoleic acid supplementation, not to a difference in VLDL production.

### Lipoprotein profile WHR effect

Our observation that the five smallest LDL fractions were significantly higher for lower body obese (WHR<1.0) subjects is somewhat surprising. At population level in the Quebec Heart Study, a weak positive correlation between WHR and LDL cholesterol is observed [30]. However, for standard clinical chemistry LDL cholesterol calculated using the Friedewald formula [31], we did not find significant differences with waist-hip-ratio. This last result shows that in the setting we are studying, the difference in the type of LDL measurement can invalidate this comparison.

### Lipoprotein profile treatment effect

The results from the lipoprotein profiles cannot be compared directly to other studies, because our study is the first to study the response of a complete lipoprotein profile to this intervention. Our observation that the lower cholesterol concentrations after linoleic acid supplementation can be attributed to changes in a large number of subfractions in the particle size range from VLDL through LDL is therefore new. However, we can compare the total cholesterol response. This response correspond well with that found by Asakura et al [32] who saw a higher fasting total cholesterol after adding MCT (medium-chain triglycerides) to the diet in various proportions for six weeks, than after four weeks of corn oil diet which contains 49% C18:2. Also Hill et al. [33] saw a reduction in fasting serum cholesterol in a long-chain fatty acid enhanced diet with 51% C18:2, but not in a MCFA enhanced diet, after only six days. A third study by Swift et al. [34] did not show differences in total cholesterol after six days of dietary MCFA versus long-chain triglyceride (with 51% C18:2) intervention. Therefore, although the difference between MCFA and linoleic acid has not been shown consistently on the short term, our longer-term results fit in well with other longer term interventions.

### Fatty Acid tracer analysis

The results of the fatty acid tracer analysis confirmed that MCFA undergo active chain elongation. The data in Figure 8 suggest that the amount of C8:0 tracer recovered in plasma is less than that of C18:2n-6, which is coherent with literature that reports an increased fat oxidation observed on diets rich in medium-chain compared to long-chain triglycerides [35], [36]. However, while St Onge and Jones 2003 [35] found adipose tissue-specific weight loss effects on a medium chain triglyceride diet, with larger loss of upper body adipose tissue, the present study did not detect differences in fatty acid metabolism between upper body obese and lower body obese subjects.

### VLDL production

From our study and from the literature, we could derive two factors that help to explain the lack of increase in VLDL production after dietary MCFA supplementation. The first are the metabolic transformations that MCFAs undergo in the liver before being released into the plasma: the active chain elongation process after dietary MCFA supplementation. This process might slow VLDL production. Secondly, studies in chicken primary hepatocites have shown that MCFAs can downregulate apoB expression, decreasing VLDL production [37], [38]. So chain elongation and downregulated apoB expression may help to explain the lack of difference in VLDL production between the two treatments.

### VLDL lipolysis and uptake

In this study we observed a higher particle lipolysis and uptake rate after linoleic acid supplementation versus MCFA supplementation. A direct comparison of these two diets in the Zucker rat, showed a lower LPL activation in the gonodal fat pad after an MCFA diet [39]; this observation is in concordance with our result. Thomas [40] saw no difference in LPL activity in humans, after a single dietary challenge, but single dietary challenge is not comparable with our longer term study. No other direct comparison is available to our knowledge. So the higher lipoprotein lipolysis and uptake rate after linoleic acid supplementation versus MCFA supplementation is consistent with observations in animals.

The observed difference in lipoprotein lipolysis and uptake can possibly be explained through PPAR-activation by linoleic acid [8]–[10]. As mentioned in the introduction, PPAR activators are known to increase LPL lipolysis and increase liver uptake of LDL particles [11], [12]. We can directly observe an increase in LPL-related lipolysis and liver uptake in the VLDL fraction. Evidence for an effect on LDL uptake is more indirect. We observe that most variation between treatments in the IDL and LDL region is found in the uptake over influx ratio instead of the HL lipolysis over influx ratio; and HL lipolysis does not respond to the treatment like LPL lipolysis does, since the HL over total lipolysis ratio changes. This indicates an increasing lipoprotein uptake process in IDL and LDL after linoleic acid supplementation. Therefore, we can conclude that our data are consistent with PPAR activation by linoleic acid.

### Potential drawbacks of the study

In this study we have been able to study the kinetics of one VLDL pool. This set-up has given information about the mechanism underlying the treatment effect, but not the mechanism underlying the difference in WHR groups. We observed a difference in LDL cholesterol subfractions between WHR groups. In order to get insight into the metabolic mechanism responsible for this difference, a study that includes LDL kinetics is necessary.

### Strong points of the study

The strong points of this study are the combination of lipoprotein profiles, the kinetic study and Particle profiler. The difference we found in VLDL kinetics between treatments could not have been quantified otherwise, except by a far more extensive kinetics study.

### Conclusions

In this study, dietary MCFA supplementation resulted in higher plasma cholesterol than linoleic acid supplementation, for nearly all VLDL, IDL and LDL subfractions in the measured lipoprotein profile. The results of the metabolic study show that VLDL lipolysis and uptake are lower for MCFA supplementation than for linoleic acid supplementation, whereas VLDL production does not differ significantly. To explain the observed differences between WHR groups a further study into LDL kinetics is necessary.

## Supporting Information

File S1 **Supporting Methods, Results, Figures and Tables.**
(PDF)
