# Supplementary material for: Dietary Medium Chain Fatty Acid Supplementation Leads to Reduced VLDL Lipolysis and Uptake Rates in Comparison to Linoleic Acid Supplementation
Source: PLoS One. 2014 Jul 21;9(7):e100376. doi: 10.1371/journal.pone.0100376 (PMC4105472; doi:10.1371/journal.pone.0100376)
Supplement: File S1 — Supporting Methods, Results, Figures and Tables. (PDF) [file pone.0100376.s001.pdf]

Dietary medium chain fatty acid supplementation leads to reduced VLDL lipolysis and uptake rates in comparison to linoleic acid supplementation

Daniël B. van Schalkwijk[1,2,3], Wilrike J. Pasman[1], Henk F.J. Hendriks[1], Elwin R. Verheij[1], Carina M. Rubingh[1], Kees van Bochove[1], Wouter H. J. Vaes[1], Martin Adiels[4], Andreas P. Freidig[1], Albert A. de Graaf[1]

Supporting Information File S1

Affiliations

Daniël B. van Schalkwijk[1,2,3]

[1]TNO, Zeist, the Netherlands and Analytical Sciences division,

[2]The Leiden Amsterdam Centre for Drug Research, Leiden, the Netherlands and

[3]The Netherlands Bioinformatics Centre (NBIC), Nijmegen, The Netherlands.

Wilrike J. Pasman[1],

[1]TNO, Zeist, the Netherlands

Henk F.J. Hendriks[1],

[1]TNO, Zeist, the Netherlands

Elwin R. Verheij[1]

[1]TNO, Zeist, the Netherlands

Carina M. Rubingh[1]

[1]TNO, Zeist, the Netherlands

Kees van Bochove[1]

[1]TNO, Zeist, the Netherlands

Wouter H. J. Vaes[1]

[1]TNO, Zeist, the Netherlands

Martin Adiels[4]

[4]Department of Mathematical Sciences, University of Gothenburg, Gothenburg, Sweden

Andreas P. Freidig[1]

1TNO, Zeist, the Netherlands

Albert A. de Graaf[1]

[1]TNO, Zeist, the Netherlands

Correspondence: Address reprint requests and correspondence to AA de Graaf, TNO, P.O. Box 360, 3700 AJ Zeist, The Netherlands. Email: albert.degraaf@tno.nl. Tel: +31 (0)88 866 50 23.

Introduction

Lipoprotein profiles were analyzed using the previously described Particle Profiler model [1,2]. This study constitutes the first application of Particle Profiler to HPLC lipoprotein profiles. The necessary data processing and adjustments in the data fitting process are described here.

Identified issues

We identified the following issues for data processing and data fitting.

1. Calibration of particle size. Different measurement methods are expected to slightly deviate in the particle size they report for any particle. The only way of calibrating the different measurement methods is through comparing the average lipoprotein composition at each particle size.
2. The HPLC method [3] returns total cholesterol and triglyceride data per subfraction with a particular particle size. However, Particle Profiler works with particle numbers, so that the HPLC data need to be converted to particle numbers.
3. Data fitting requires assigning weights to data points that allow the model to optimally adjust itself to the dataset.

Biochemical composition and lipoprotein size

The HPLC lipoprotein profile data measured by Liposearch [3] contain total cholesterol (TC) and triglyceride (TG) contents per lipoprotein subfraction. Therefore, the only possible comparison between the biochemical composition of this lipoprotein profile and the particle composition model of Tuzikov *et al.* [4], used for constructing Particle Profiler, was to compare the TG/TC ratio in each lipoprotein subfraction. Therefore, we used the TG/TC ratio to calibrate the particle size difference between the HPLC method and the model.

The results of the TG/TC comparison are shown in Supporting Figure 1. In the initial situations, a deviation was found between the TG/TC ratios of the HPLC method and the Tuzikov model. However, this discrepancy could be remedied satisfactorily by shifting the particle size of the Tuzikov model to approximately 4.9 nm larger than

originally reported. Therefore, based on a comparison of biochemical composition, we could carry out a reasonable calibration of measured and modeled particle sizes.

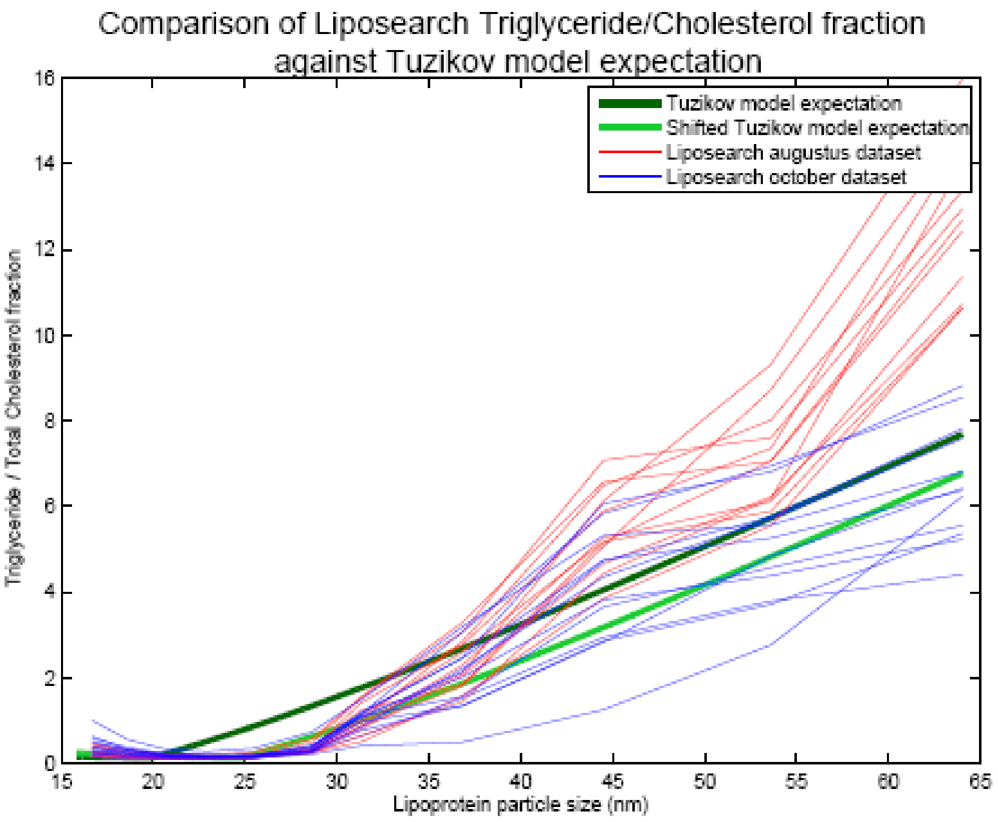

Supporting Figure S1: Comparison of Liposearch data with the Tuzikov biochemical lipoprotein particle composition model, without and with incorporation of the particle size shift. At the LDL and IDL range, the shift was fitted at 4.9 nm.

Conversion to Particle Numbers

The HPLC measurement method returns the triglyceride and cholesterol concentration in each lipoprotein subfraction. Particle Profiler works with particle concentrations, which therefore need to be calculated from the available data. This calculation proceeds as follows. Supporting Table 1 explains the mathematical notation.

First, the core volume of an average particle in a certain subclass is calculated assuming a spherical shape, the size of the particles in a subclass is known and the size of the particle shell is also known [5].

$$V_{core} = \frac{\pi}{6} \cdot (L_d - L_s)^3$$

Second, the volume fraction of triglycerides in the core is calculated, knowing that triglycerides and cholesterol esters are only present in the core. Free cholesterol is present in the particle shell. In order to devide the measured total cholesterol in free and esterified cholesterol, the average ratio for particles at that size is used, taken from [4].

$$f_{v,tg,core} = \frac{\frac{C_{tg}}{\rho_{tg}}}{\frac{C_{tg}}{\rho_{tg}} + \frac{C_{tc} \cdot \frac{m_{ce}}{m_{fc} + m_{ce}}}{\rho_{ce}}}$$

The triglyceride and cholesterol mass in each particle is calculated from the results of the preceding calculations.

$$m_{core,tg} = f_{v,tg,core} \cdot V_{core} \cdot \rho_{tg}$$

$$m_{core,ce} = (1 - f_{v,tg,core}) \cdot V_{core} \cdot \rho_{ce}$$

$$m_{part,fc} = m_{core,ce} \cdot \frac{m_{fc}}{m_{ce}}$$

$$m_{part,tc} = m_{core,ce} + m_{part,fc}$$

From both the calculated triglyceride and total cholesterol content per particle and the measured triglyceride and total cholesterol concentration in a certain subclass, the particle concentration is calculated. If the calculation proceeds correctly, these two particle concentrations should be identical.

$$C_{particles,tg} = \frac{C_{tg}}{m_{core,tg}}$$

$$C_{particles,tc} = \frac{C_{tc}}{m_{core,tc}}$$

$$C_{particles} = C_{particles,tc} = C_{particles,tg}$$

Supporting Table S1: Mathematical Notation

Symbol	meaning
V_{core}	Volume of the particle core
L_d	Length measure of particle radius
L_s	Length measure of particle shell
$f_{v,tg,core}$	Fraction of volume in particle core taken up by triglycerides
C_*	Concentration of constituent * in plasma, in the lipoprotein subclass under consideration.
ρ_*	Density of constituent *
m_*	Mass of constituent *
subscripts	*tg* – triglycerides
	tc – total cholesterol
	ce – cholesterol esters
	fc – free cholesterol
	core – refers to particle core
	part – refers to whole particle
	particles, tg/tc – particles in plasma, calculated using triglycerides or total cholesterol.

Weights

Fitting the model to HPLC data requires a good adjustment of the 'weights' that determine the importance of deviations between measurements and data for each data point. Especially the small and medium VLDL pools should really be fitted exactly right, since they are essential for a right characterization of the VLDL metabolism ratios. The two larger LDL pools are important for quantifying the lipolysis flux from VLDL to LDL. The largest VLDL pools were not given great importance, as the model was developed with datasets of lipoprotein kinetics for which only two VLDL pools are available. To be able to interpret this large VLDL pool correctly, more detailed kinetics datasets are necessary, which are not available at this time. The square roots are inserted since the distance measure between lipoprotein profile data and model outcomes is a sum of squares measure. The weights used for fitting the data are given in Supporting Table 2.

Supporting Table S2: weights used for fitting model to data

Fraction name	Weight
Extra small LDL 1	1
Extra small LDL 2	$\sqrt{2}$
Extra small LDL 3	$\sqrt{2}$
Small LDL	$\sqrt{2}$
Medium LDL	$\sqrt{5}$
Large LDL	$\sqrt{10}$
Small VLDL	$\sqrt{100}$
Medium VLDL	$\sqrt{100}$
Large VLDL 1	$\sqrt{100}$
Large VLDL 2	$\sqrt{0.1}$
Large VLDL 3	$\sqrt{0.1}$

ApoB and lipoprotein kinetics

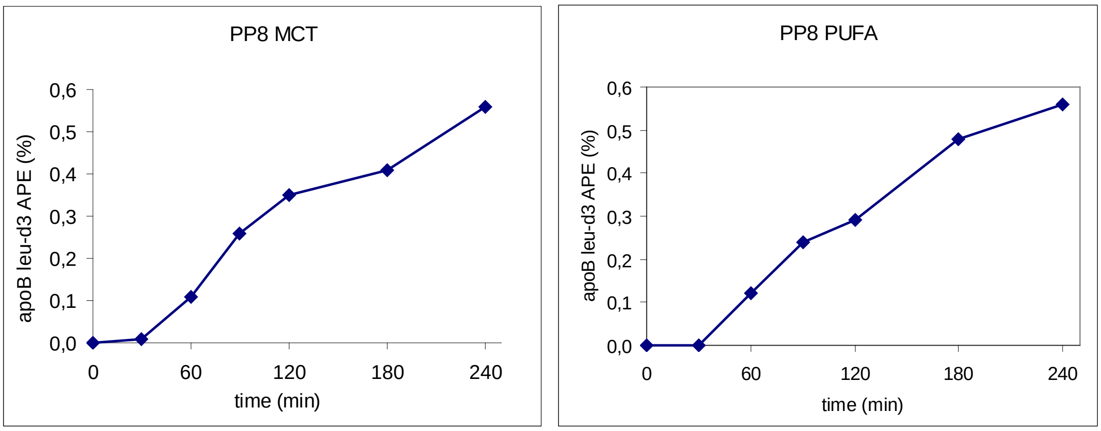

Supporting Figure S2. Typical apoB d3-leucine enrichment time course after both treatments.

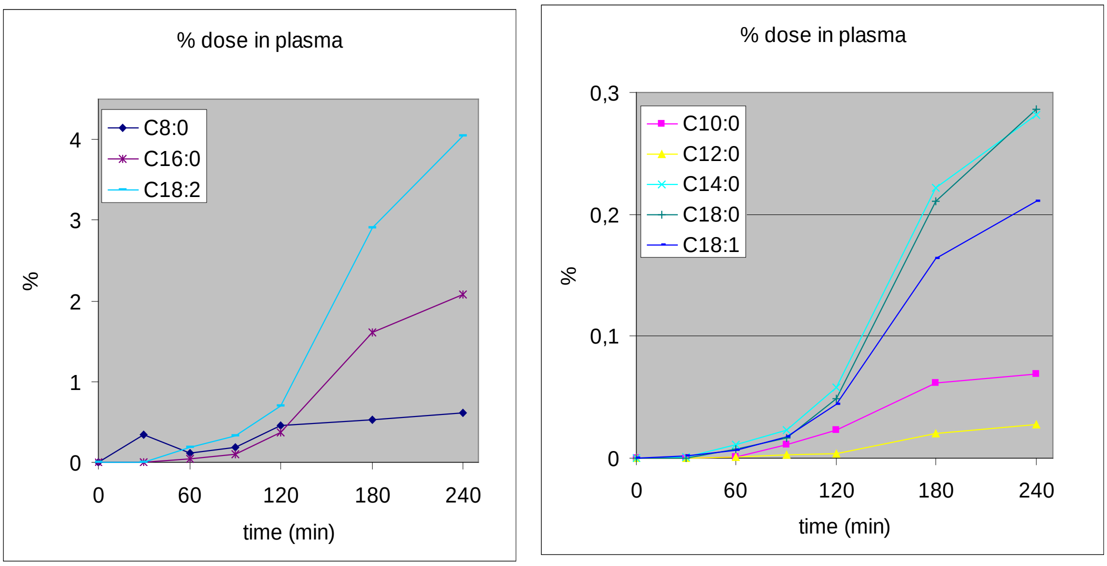

Supporting Figure S3. Representative postprandial time courses of ^{13}C label in plasma fatty acids from an individual of the high WHR group that received the MCT dietary fat supplementation.

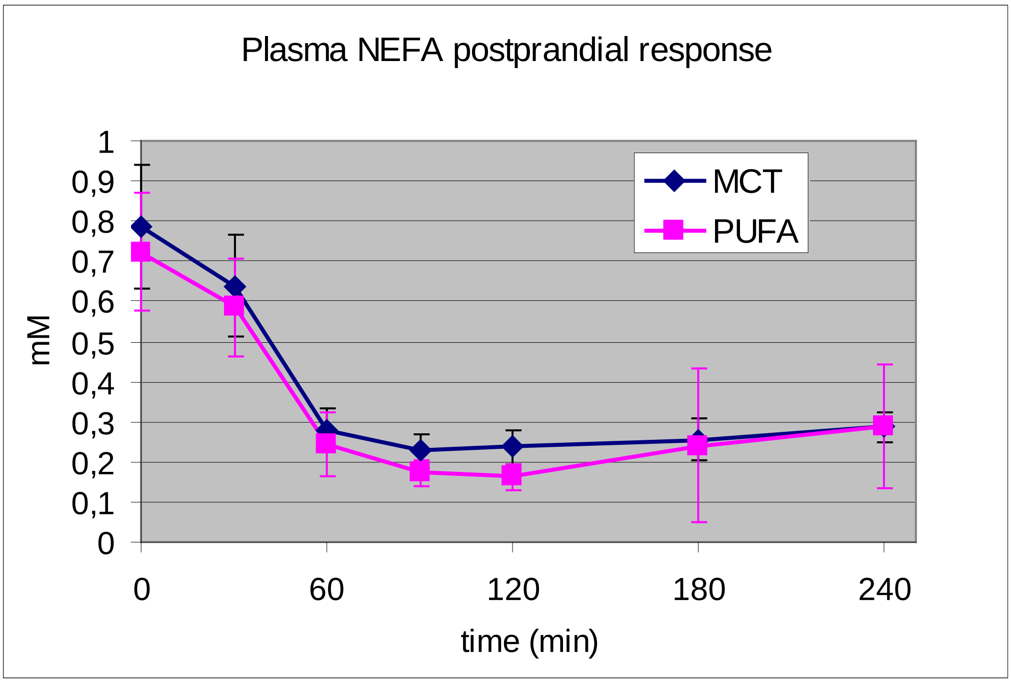

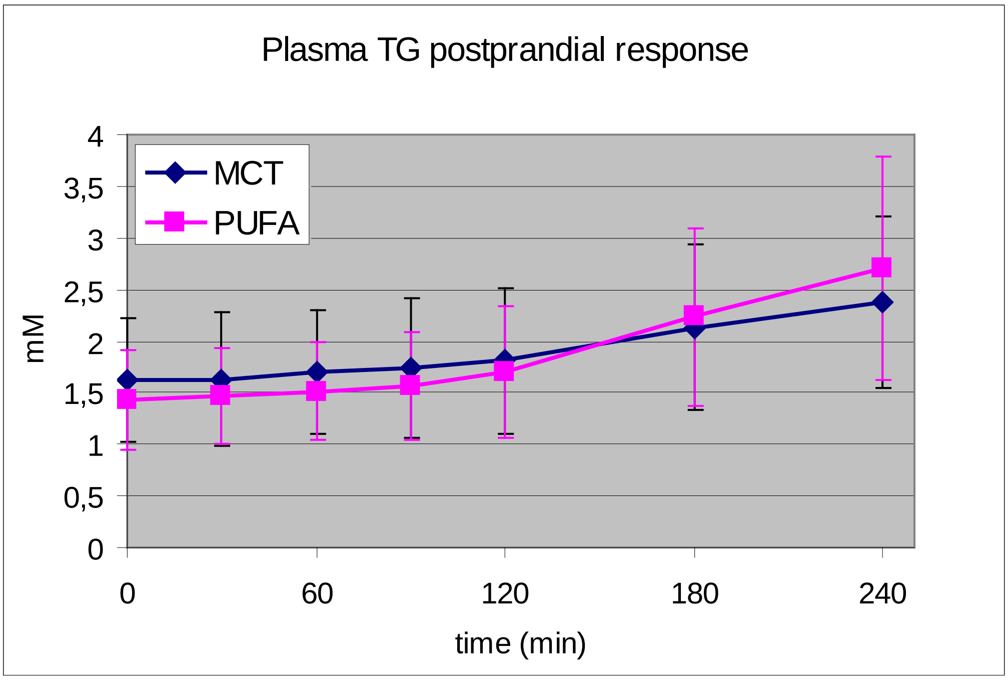

Supporting Figure S4. Average postprandial time courses of NEFA and triglycerides after both MCT and PUFA supplementation.

References

1 van Schalkwijk DB, de Graaf AA, van Ommen B, van Bochove K, Rensen PCN et al. (2009) Improved cholesterol phenotype analysis by a model relating lipoprotein life cycle processes to particle size. J Lipid Res 50: 2398-2411.

2 van Schalkwijk DB, van Bochove K, van Ommen B, Freidig AP, van Someren EP et al. (2010) Developing computational model-based diagnostics to analyse clinical chemistry data. Brief Bioinform 11: 403-416.

3 Usui S, Hara Y, Hosaki S, Okazaki M (2002) A new on-line dual enzymatic method for simultaneous quantification of cholesterol and triglycerides in lipoproteins by HPLC. J Lipid Res 43: 805-814.

4 Tuzikov FV, Tuzikova NA, Galimov RV, Panin LE, Nevinsky GA (2002) General model to describe the structure and dynamic balance between different human serum lipoproteins and its practical application. Med Sci Monitor 8: MT79-88.

5 Shen BW, Scanu AM, Kezdy FJ (1977) Structure of human serum lipoproteins inferred from compositional analysis. Proc Natl Acad Sci U S A 74: 837-841.
